# Self-Assembly of Curved Photonic Heterostructures by the Hanging Drop Method

**DOI:** 10.3390/polym18080924

**Published:** 2026-04-09

**Authors:** Ion Sandu, Claudiu Teodor Fleaca, Florian Dumitrache, Iuliana Urzica, Iulia Antohe, Marius Dumitru

**Affiliations:** Lasers Department, National Institute for Lasers, Plasma and Radiation Physics, 409 Atomistilor Street, 077125 Magurele, Romania; ion.sandu@inflpr.ro (I.S.); claudiu.fleaca@inflpr.ro (C.T.F.); florian.dumitrache@inflpr.ro (F.D.); iuliana.iordache@inflpr.ro (I.U.)

**Keywords:** curved photonics, hanging-drop self-assembly, photonic heterostructures, order–disorder coupling, 2.5D metasurfaces

## Abstract

By combining hanging-drop self-assembly with melt infiltration and selective inversion, we fabricate millimetric and free-standing curved photonic heterostructures that integrate infiltrated-opal, inverse-opal, embossed, and white-scattering 2.5D metasurface domains within a single continuous body. These architectures enable configurations inaccessible to planar fabrication, including naturally formed concavities within convex inverse-opal films and alternating ordered/single-layer regions that preserve local coherence while introducing disorder at larger scales. Across these heterogeneous curved landscapes, we observe optical phenomena absent in flat photonic structures—spectrally selected lateral collimation, geometry-shifted ghost images, and transmission-derived valleys shaped by curvature-mediated Bragg extraction. Their origin lies in the geometric constraints inherent to curved assemblies, where spatially varying normals, non-parallel lattice orientations, and topologically required defects couple order and disorder into a distributed-coherence regime. This coupling expands the accessible photonic state space, establishing curvature as an active functional degree of freedom rather than a geometric constraint, positioning the self-assembled photonic heterostructures as a scalable route toward multifunctional 3D metasurfaces and new regimes of light–matter interaction.

## 1. Introduction

The vision of controlling light with artificial structures dates back to the seminal works of Yablonovitch and John (1987), who independently introduced the concept of photonic band gaps as the optical analogues of electronic band structures in semiconductors [1,2]. Since then, the field of photonics has developed into a multidisciplinary arena, integrating condensed matter physics, nanofabrication, chemistry, and materials science. Structural color in natural systems—such as butterfly wings, peacock feathers, and natural opals—offered the first demonstration that periodic dielectric arrangements can manipulate light through interference and diffraction [3,4]. Artificially replicating such effects has fueled advances in telecommunications, optical sensing, and energy technologies, and continues to inspire the design of photonic crystals and metamaterials [5]. In this sense, polymers play a central role in many emerging photonic systems due to their processability, low costs, tunable refractive index, possibility to be functionalized for biological essays and compatibility with large-scale fabrication. In particular, polymer-templated inverse opals and polymer infiltrated photonic structures provide a versatile platform for constructing three-dimensional photonic architectures with controllable optical response.

Historically, progress in photonic crystal fabrication has been driven by top-down methodologies. Lithography [6], chemical etching [7], and sequential deposition [8] have delivered unparalleled precision, scalability, and compatibility with CMOS technology, underpinning the success of integrated optics and optoelectronics. These deterministic routes enabled the development of two-dimensional photonic slabs, metasurfaces, and resonators, with applications ranging from filters and waveguides to bound states in the continuum (BICs) [9] and high-performance diffractive films [10]. Yet their strength—planar precision—also defines their central limitation: the confinement to thin, two-dimensional architectures. Even the most sophisticated devices operate within micrometer-thick domains, advancing technical performance while reinforcing geometric restrictions. 

In parallel, bottom-up approaches emerged as a complementary strategy. Inspired by colloidal physics and soft matter science, methods such as self-assembly [11,12], inverse opal templating [13], and co-assembly [14] exploit the spontaneous ordering of colloidal particles into periodic lattices. These techniques promise low-cost, large-area fabrication and access to truly three-dimensional periodicity. However, despite remarkable progress, bottom-up photonics has remained largely restricted to planar films deposited on substrates. This reliance on flat geometries has limited the development of volumetric photonic crystals with interconnected porosity, omnidirectional photonic band gaps, and tunable curvature. The persistence of two-dimensionality reflects not only technical but also epistemological biases. The field has long privileged deterministic, design-driven fabrication while relegating self-assembly to a peripheral role. As a result, bottom-up methods are often perceived as exploratory or decorative, despite their proven ability to generate highly ordered, multifunctional structures [15]. 

Recent innovations in artificial intelligence-assisted assembly [12], microfluidic templating [16], and responsive co-assemblies [17] highlight the growing sophistication of bottom-up design. Yet the critical challenge remains: to transition from quasi-2D demonstrations to functional three-dimensional photonic architectures that can rival or complement top-down counterparts. The fabrication of curved colloidal photonic crystals has been reported in spherical [18,19,20], cylindrical [21], and toroidal [22] geometries, but their dimensions typically remain confined to tens of micrometers, rarely exceeding a few hundred. These size constraints limit their capacity to sustain omnidirectional band gaps or to function as macroscopic optical components. Moreover, most studies focus on synthesis rather than integration, leaving open the challenge of embedding curved colloidal crystals directly into practical photonic systems. Recent trends highlight the urgency of addressing this gap. Three-dimensional photonic crystals are increasingly sought for applications in biosensing, where environmental refractive-index shifts translate into optical signals [23]; in energy, where periodic porosity enhances catalytic and storage processes [24]; and in communications, where omnidirectional band gaps and diffractive control could advance optical routing [25]. In particular, lab-on-fiber platforms are rapidly evolving as compact and powerful technologies for in-situ diagnostics and environmental monitoring. For instance, Dolci et al. (2025) demonstrated a fiber-tip photonic crystal biosensor capable of real-time, referenced detection in serum [26], while Ramola et al. (2025) reviewed recent advances in photonic crystal fiber-based plasmonic biosensors with applications in biology and medicine [23]. Similarly, Wang et al. (2024) reported a three-dimensional photonic crystal gas sensor with ultrafast response and trace-level detection [27], and Rafiee et al. (2025) highlighted how coupled resonator designs dramatically improve refractive-index sensitivity [28]. These examples emphasize that the integration of volumetric photonic crystals into practical platforms is both feasible and urgently needed.

Within this context, the hanging-drop method has emerged as a distinctive self-assembly route that overcomes several limitations of conventional techniques [29,30,31,32]. Unlike vertical deposition [33], spin-coating [34], sedimentation [35], or evaporation-driven self-assembly on substrates [36], which primarily generate supported planar films rather than free-standing curved architectures, the hanging-drop approach exploits the curved liquid–air interface of a suspended droplet to direct colloidal ordering. The curvature-driven confinement distributes assembly stresses more uniformly, thereby reducing defects and enabling ordered growth along non-planar geometries. As the solvent evaporates, colloidal particles organize into large, free-standing crystals with convex, ellipsoidal, paraboloidal, or hyperboloidal morphologies (see Refs. [30,31,32] for schematic representation of the assembly mechanism). Such morphologies are difficult to achieve by other self-assembly routes, and extend the design space beyond flat substrates. The potential of this method is significant. Volumetric colloidal crystals several hundred micrometers to millimeters in size exhibit enhanced optical responses compared with their planar counterparts. Sufficient thickness and periodicity support complete photonic band gaps [37], while curved morphologies suppress boundary-related losses [38] and enable isotropic structural coloration [39]. Unlike thin films, these large-scale crystals exhibit robust Bragg diffraction, reproducible optical signatures, and improved mechanical stability, making them viable candidates for integration into functional photonic devices.

In this study, we extend the hanging-drop approach toward the fabrication of curved photonic heterostructures. From a structural perspective, optical heterostructures consist of multiple photonic domains with distinct lattice and/or dielectric properties integrated within a single continuous architecture, enabling interactions between them. In this context, we realize free-standing, millimetric structures that integrate infiltrated-opal and inverse-opal domains within the same macroscopic architecture. These systems combine diffraction, reflection, and curvature-induced optical confinement within geometries that cannot be achieved by planar fabrication. Structural and optical characterization—through optical and scanning electron microscopy together with retro-reflection spectroscopy—confirms the formation of ordered nanostructures exhibiting reproducible and direction-dependent photonic responses. The results position the hanging-drop route as a practical and versatile bottom-up alternative for constructing three-dimensional photonic architectures, bridging the gap between self-assembly and functional device integration. Polystyrene serves both as structural scaffold and functional optical medium, enabling the formation of large curved photonic heterostructures through melt infiltration and template inversion.

## 2. Materials and Methods

### 2.1. Materials

The following materials were used in all experiments: SiO_2_ submicron spheres (0.264 µm diameter, 5% *w*/*v* aqueous suspension; microParticles GmbH, Berlin, Germany); polystyrene flakes (Sigma-Aldrich, St. Louis, MO, USA, Product No. 331651, melting point 240 °C, typically irregular fragments of ~4–5 mm lateral size, ~70 mg per flake); reagent-grade ethanol and hydrofluoric acid (HF); and glass rods (5 mm diameter).

### 2.2. Methods

#### 2.2.1. Fabrication of Polystyrene Ellipsoidal Substrates

A 5 mm glass rod was fixed horizontally in a furnace at 250 °C. A polystyrene flake was briefly touched to the rod using tweezers, softened, adhered, and formed a molten pendant droplet. The droplet self-separated into a falling portion and a stable portion that remained attached. Upon cooling, this produced a reproducible semi-ellipsoidal substrate (~10 × 5 × 1.2 mm; ~40 mg), independent of the initial flake mass due to the self-limiting nature of the process. Heat-protective equipment was used throughout.

#### 2.2.2. Self-Assembly of SiO_2_ Colloidal Opals

A ~6 µL droplet of SiO_2_ colloidal solution mixed with ethanol (5:1 *v*/*v*) was deposited onto the polystyrene substrate using a syringe, followed by 2–3 additional droplets (~6 µL each) to enlarge the volume. Ethanol improved wetting and lateral spreading. Droplets dried under ambient laboratory conditions for 60–120 min, forming an iridescent ellipsoidal colloidal crystal.

#### 2.2.3. Melt Infiltration of the Colloidal Crystal

The molten polystyrene does not only stabilize the colloidal lattice but also defines the final refractive-index landscape of the photonic structure, transforming the fragile silica opal into a mechanically robust polymer photonic architecture.

The furnace temperature was set to 230 °C, 10–20 °C lower than in Section 2.2.1, to minimize cracking induced by contact-angle fluctuations. The sample was held for 10–15 min to allow molten polystyrene infiltration, then cooled naturally, yielding a mechanically robust infiltrated opal anchored to the supporting rod.

Under nominally identical macroscopic conditions, the process exhibits a statistically reproducible variability rather than strict determinism. Small fluctuations in cooling rate or interfacial tension can result either in intact films or in localized fissures. Empirically, maintaining all thermal stages at a single temperature (T_0_) consistently produces multiple cracks, whereas a two-step thermal profile (T_0_ → T_1_) restricts fracturing to a single fissure aligned with the major, or less frequently the minor, optical axis. When the temperature difference between T_0_ and T_1_ is kept below 20 °C, crack formation is suppressed. These observations indicate that residual stress arises predominantly from contact-angle hysteresis during infiltration and solidification, rather than from the infiltration process itself.

#### 2.2.4. Transfer of Molten Polystyrene to the Opal Pole

A new molten polystyrene droplet was formed at 250 °C. The infiltrated opal was touched to its base, transferring a controlled amount of melt to the opal pole, which solidified into a stable polystyrene cap upon cooling.

#### 2.2.5. Formation of Hemispheres and Spherical Caps

The assembly was placed hanging horizontally in a furnace at 150–180 °C. Within 3–4 min, the transferred polystyrene softened into a 2.5–3.0 mm hemisphere, then gradually evolved into a flattened spherical cap a few hundred micrometers high. The sample was visually monitored and removed once the desired curvature was reached.

#### 2.2.6. Control of Gas Bubbles

During hemispherical-cap formation, 50–100 µm gas bubbles may form. Their density increases with temperature and residence time. In shallow caps, bubbles typically vanish through degassing.

#### 2.2.7. Infiltrated-Opal/Inverse-Opal Heterostructures

A second colloidal layer was created by applying 2–3 droplets of SiO_2_ suspension onto the spherical-cap pole, following Section 2.2.2. This layer was infiltrated at 200 °C (Section 2.2.3). After cooling, silica spheres were removed using 25% aqueous HF, via:

(a) Localized HF-drop dissolution:
  A droplet of HF was placed at the pole for 10–15 min, then replaced with water for dilution. The sample could remain on the rod or be detached using a steel blade.
(b) Full immersion:
  The substrate was first detached, then immersed in diluted HF for 10–15 min, rinsed thoroughly, and dried. All HF-handling followed institutional safety protocols with HF-resistant gloves and protective eyewear.

#### 2.2.8. White-Scattering Metasurface Synthesis

A single infiltrated opal (Section 2.2.3) was subjected to localized HF dissolution (Section 2.2.7 (a). A new SiO_2_ colloidal crystal was grown on the resulting inverse-opal surface, infiltrated again, and subjected to a second silica dissolution. The metasurface was then detached from the rod.

#### 2.2.9. Embossed White-Scattering Metasurfaces

A convex infiltrated opal (on an ellipsoid or spherical cap) was infiltrated at 135 °C for 15 min, inducing viscoelastic embossing. Silica was removed in HF, followed by rinsing, drying, and detachment.

### 2.3. Reproducibility

The fabrication procedures are highly robust and exhibit self-limiting, self-stabilizing behavior, ensuring reproducibility even when input parameters vary within broad ranges. Critical parameters are limited to furnace temperatures (250 °C, 230 °C, 150–180 °C, 135 °C; tolerance ±5–10 °C) and approximate residence times. All other parameters—including the initial flake mass, droplet number (typically 2–3), droplet volume (±1–2 µL), and minor variations in drying time—are non-critical and do not measurably affect geometry or optical response.

The system self-selects the stable polystyrene volume that remains attached to the glass rod, yielding nearly identical ellipsoidal substrates regardless of starting mass. Hemispherical caps likewise converge to reproducible curvatures determined by surface-tension equilibration. Geometric variations remain within 5–10%, and all spectral signatures are reproduced across at least three independent samples for each structure type. The rationale behind the chosen protocol follows a non-systematic methodology previously formulated as part of a broader inquiry into coherence and control in complex systems [40].

### 2.4. Investigations

Macroscopic morphology was assessed using zoom-camera imaging and optical microscopy. High-resolution SEM images were obtained using a Thermo Fisher Scientific Apreo S system, Auburn, AL, USA. UV–Vis reflectance spectra were recorded using an Avantes AvaLight-DHc light source and an AvaSpec-ULS2048CL-EVO, Apeldoorn, The Netherlands, spectrometer, coupled through a 6-around-1 fiber probe (six illumination fibers surrounding a central collection fiber).

### 2.5. Generative Artificial Intelligence Contribution

Figure 5a was produced with the assistance of ChatGPT 5 (OpenAI) and refined by the authors to ensure alignment with experimental data.

## 3. Results and Discussion

### 3.1. Morphological and Structural Results

**Figure 1:**
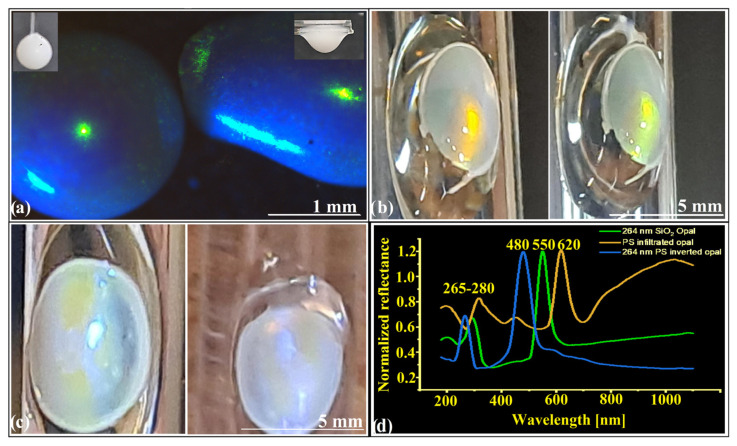
(**a**) Optical microscopy of semispherical and ellipsoidal SiO_2_ opals (d = 264 nm). Insets: corresponding hanging drops; (**b**) Polystyrene-infiltrated opal from different angle; (**c**) Inverse opal from different angles; (**d**) UV–Vis retro-reflection spectra of silica opal, infiltrated opal, and inverse opal. Images in (**b**,**c**) were acquired under oblique observation (≈30–60° relative to the surface normal) to enhance structural color visibility.

The hanging-drop [30,31,32] method exemplifies bottom-up fabrication at its most efficient: a simple yet precise route to three-dimensional colloidal photonic crystals, unconstrained by planar substrates. Unlike top-down lithographic strategies—intrinsically bound to two-dimensional architectures—the hanging-drop method produces large curved crystals in which symmetry and optical behavior follow the geometry imposed by the liquid–air interface. It requires no cleanroom, no specialized equipment, and tolerates fluctuations in temperature, humidity, vibration, and air currents. Crystals form equally well from silica or polystyrene spheres, across nanometric to micrometric scales. The sole prerequisite is monodispersity—long recognized as the structural condition linking order to optical coherence. A droplet of colloidal suspension is formed at the tip of a syringe and suspended beneath a support. During 1–2 h of natural evaporation, the spheres self-assemble into ordered close-packed arrays. Once dry, the crystal can be detached, displaying vivid structural color under white light, provided the sphere diameter lies within the photonic range. Morphology depends on droplet orientation: hemispherical when grown from a vertical fiber [31], ellipsoidal when supported on a horizontal rod [32] (Figure 1a). Sizes range from 1 to 5 mm, governed by droplet volume, concentration, and substrate geometry.

Structurally, these crystals display high-quality ordering both at the surface and throughout the bulk. Domains span tens of micrometers, forming hundreds of hcp layers with excellent mechanical resilience—ideal for direct handling and integration. The only defect-prone region lies at the liquid–substrate interface, where incomplete packing or voids appear. Yet these self-assembled opals can be further stabilized and functionalized by infiltration with melts or polymer solutions, producing infiltrated opals (Figure 1b). Infiltration thus acts not merely as a consolidation step, but as a gateway to structural metamorphosis—transforming a fragile colloidal lattice into a robust photonic medium. Subsequent removal of the colloidal template (via HF for silica or selective solvents for polystyrene) yields inverse opals (Figure 1c), extending structural hierarchy and refractive contrast. Both infiltrated and inverse architectures exhibit strong angular dependence and directional optical response. UV–Vis retro-reflection spectra (Figure 1d) reveal sharp photonic bands from the (111) planes, in full agreement with Bragg diffraction, confirming long-range periodicity. Comparative measurements across curved and nominally planar regions consistently show a blue shift in the (111) band—on average ~20 nm—accompanied by apparent reflectance values exceeding 100%. These deviations stem not from increased efficiency, but mainly from geometry: curvature induces angular integration of Bragg conditions, while simultaneously collecting and redirecting light from a broader solid angle into the detection fiber [31,32]. As a result, the spectrum reflects the curvature-imposed angular average rather than a single Bragg angle. 

**Figure 2:**
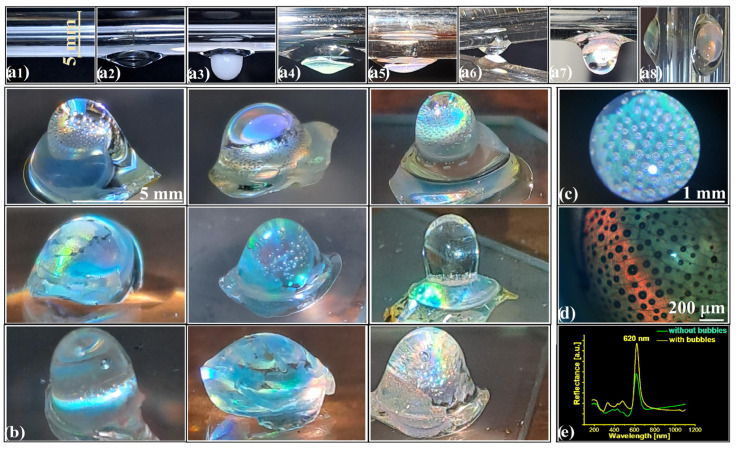
(**a1**–**a8**) Key steps in convex infiltrated-opal/hemisphere synthesis; (**b**) Examples of convex heterostructures; (**c**,**d**) Gas-bubble arrays inside the polystyrene hemisphere; (**e**) Retro-reflection spectra at the hemisphere pole.

Because all three architectures—opal, infiltrated, and inverse—share a common geometric ancestry, they can be sequentially combined to form hierarchically coupled domains, each retaining optical coherence while contributing distinct refractive landscapes. When integrated through compositional or geometric interfaces, these self-assembled materials evolve into functional optical architectures exhibiting emergent behaviors inaccessible to planar systems. In practice, fabrication remains strikingly minimal. A 5 mm glass rod suspended in a 250 °C furnace (Figure 2(a1)) melts polystyrene into a hanging droplet, which solidifies into an ellipsoidal substrate (~10 × 5 × 1.2 mm, Figure 2(a2)).

This curved surface supports the self-assembly of silica spheres (Figure 2(a3)), yielding a convex opal (Figure 2(a4)). Upon reheating, the polystyrene remelts and infiltrates the opal lattice (Figure 2(a5)), while a second droplet added at the pole forms a hemispherical meniscus lens (Figure 2(a6)). The resulting structure is a fully integrated optical element in which an infiltrated-opal mirror is seamlessly coupled to a converging polymer lens (Figure 2(a7)). Once detached from the supporting rod, the heterostructure behaves as a freestanding component capable of simultaneous focusing, filtering, and reflection (Figure 2(a8)). Strong, angle-dependent iridescence is observed across the curved surface (Figure 2b). Within the polymeric lens, gas bubbles with diameters of 50–100 µm nucleate spontaneously during solidification (Figure 2c,d) without compromising mechanical stability or shifting the (111) photonic band position (Figure 2e). This structural configuration is compatible with mechanically or optically tunable responses, consistent with earlier observations of acousto-optic and photoelastic effects at polymer–air interfaces [41,42].

**Figure 3:**
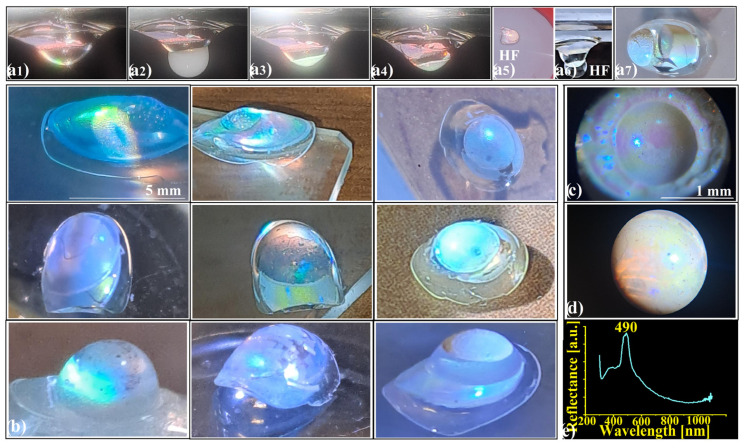
(**a1**–**a7**) Key steps in convex infiltrated-opal/inverse-opal synthesis; (**b**) Examples of convex heterostructures; (**c**,**d**) Microscopies of metasurfaces obtained at 250 °C and 200 °C; (**e**) Retro-reflection spectrum of the heterostructure.

This pathway of controlled complexity scales naturally. A convex infiltrated opal bearing a hemispherical polystyrene lens at its pole (Figure 3(a1)) can be extended by depositing an additional colloidal droplet on the hemisphere’s apex (Figure 3(a2)).

The droplet self-assembles into a secondary opal layer (Figure 3(a3)) that, after infiltration (Figure 3(a4)) and selective silica removal (Figure 3(a5,a6)), transforms into an inverse-opal metasurface (Figure 3(a7)). Silica spheres can be removed either globally—across the entire heterostructure except for the buried opal–lens interface—by immersion in aqueous hydrofluoric acid (Figure 3(a5)), or locally, by applying a reactive droplet directly onto the lens surface (Figure 3(a6)). The coverage of the secondary opal layer depends sensitively on geometry. Full coverage occurs only for flattened lenses, achievable by tuning the volume of the transferred polystyrene droplet, the temperature, and the duration of the thermal treatment of the starting structure (Figure 3(a1)). Such optimization extends the process toward metasurface-type heterostructures, in which the hemisphere height is reduced to several hundred micrometers. This geometry eliminates bubble formation entirely while promoting uniform infiltration and surface patterning. Conversely, when the secondary structure retains its semi-spherical form, a transparent window persists at the ellipsoid–lens interface, allowing oblique light to access the underlying infiltrated opal directly. Conversion into an inverse opal narrows the reflected color range from yellow–violet (infiltrated opal) to blue–violet (inverse opal) (Figure 3b), while simultaneously increasing diffraction intensity. This contrast enhancement arises from the inversion of the refractive index topology—from a high-index continuous phase (silica) to a low-index one (air)—which amplifies scattering efficiency and tightens spectral selectivity. Optical microscopy images of the hemispherical metasurface (Figure 3c,d) reveal clear differences in diffracted color and angular distribution when different processing temperatures are applied (250 °C in Figure 3c and 200 °C in Figure 3d), likely correlated with the metasurface thickness. However, the corresponding UV–Vis spectra (Figure 3e) consistently exhibit a single photonic band centered near 490 nm, associated with the inverse-opal layer. These observations mark the transition from deterministic fabrication toward a regime where optical response is governed by hierarchical screening and interfacial dominance. The absence of the (111) band of the infiltrated opal (620 nm) could result from several non-exclusive causes: (i) partial inversion near the ellipsoid–hemisphere interface, which may attenuate or obscure the underlying (111) reflection; (ii) a metasurface sufficiently thick to optically screen the substrate response; or (iii) the strong refractive-index contrast between the two domains, which can produce a substantial imbalance in reflected intensities. Experimental immersion of simple opal–lens heterostructures such as those in Figure 2 into an aqueous HF bath confirms that the interface remains mechanically intact, suggesting that the first hypothesis—partial inversion near the ellipsoid–hemisphere interface—is not plausible. However, SEM investigation is required to conclusively distinguish among the last two scenarios.

**Figure 4:**
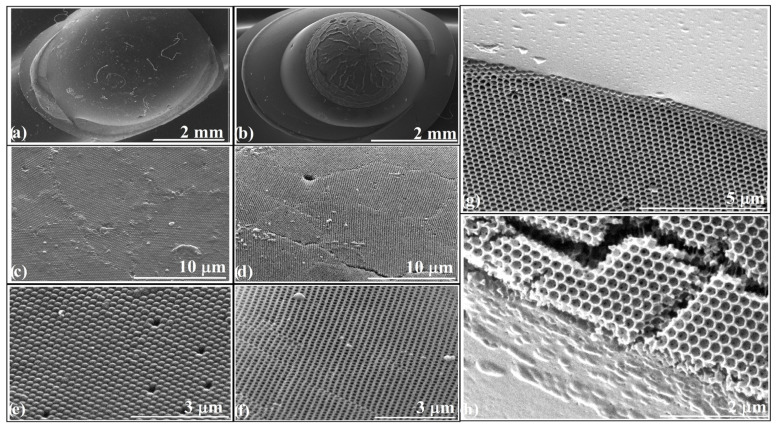
SEM images of: (**a**) Infiltrated opal with hemisphere and gas bubbles; (**b**) heterostructure metasurface: (**c**,**d**) Large-area top views; (**e**,**f**) Close-ups; (**g**) Inflection region between ellipsoid and hemisphere; (**h**) Cross-section of inverse opal. The cross-section in (**h**) was obtained by mechanical fracture of the sample to expose the internal structure.

In Figure 4, we summarize the morphological and structural features that define the curved photonic heterostructures and underpin their optical response. At this stage, it is important to emphasize that the observed optical behavior arises not from enhanced Bragg efficiency, but from curvature-mediated redistribution of coherent optical pathways.

Figure 4a shows the infiltrated-opal domain combined with a hemispherical polystyrene lens, while Figure 4b depicts the corresponding structure in which the hemisphere has been transformed into an inverse-opal metasurface. Together, they illustrate the transition from a purely refractive geometry to one that combines refraction and diffraction within the same curved body. Figure 4c,d present top-view SEM images of the infiltrated-opal and inverse-opal components, revealing highly ordered domains extending over hundreds of micrometers with minimal macroscopic defects. At higher magnification, Figure 4e,f emphasize local surface continuity and uniformity, where minor dislocations and voids appear without disrupting the global periodicity of the photonic lattice—a remarkable level of order for a structure assembled through evaporation-driven and melt-infiltration self-organization. The optical interaction regions are identified in Figure 4g,h. Figure 4g captures the smooth inflection zone between the curved surface of the ellipsoid and the base of the polystyrene hemisphere—a region critical for optical continuity and energy transfer across domains. Figure 4h displays a cross-section through the inverse-opal metasurface, showing a uniform layer thickness of approximately 3–5 periods. This structural precision allows us to rule out metasurface overgrowth as the cause for the disappearance of the infiltrated-opal (111) band in the corresponding UV–Vis spectrum (Figure 3e); thus the large refractive-index contrast between the two domains remains as the cause that produces the strong imbalance in reflected intensities.

### 3.2. Optical Phenomena and Light–Geometry Interaction

**Figure 5:**
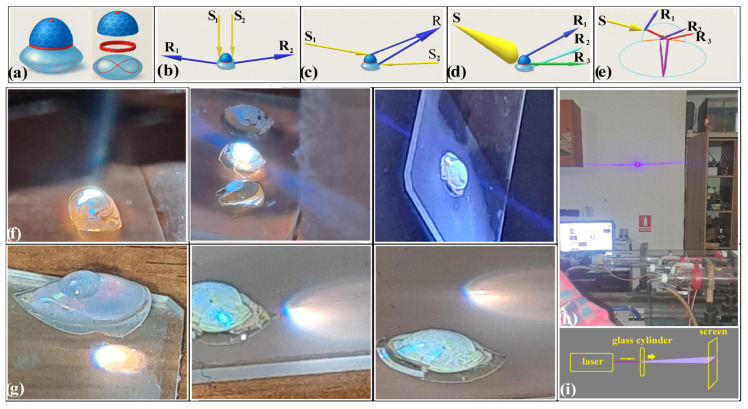
(**a**) Optically active regions; (**b**–**e**) Main light–structure interaction regimes; (**f**,**g**) Representative examples: (**f**) broadband white-light illumination showing lateral collimated blue beam; (**g**) off-focus illumination with a converging lens showing ghost-like secondary images; (**h**) 90° laser deflection by a glass rod; (**i**) Beam-redirection schematic. (S: incident beam, R: reflected beam).

With the morphological continuity now established (Figure 4), focus shifts from how the structure forms to how it acts. In this context, curvature-driven photonics denotes regimes in which spatially varying surface normals and non-parallel lattice orientations actively select, redistribute, and couple optical modes. As a result, coherence is preserved but redistributed by curvature, giving rise to optical behaviors inaccessible to planar systems. In a convex–convex photonic heterostructure consisting of semi-ellipsoidal and hemispherical polystyrene–air inverse-opal domains, four optically active points independent of illumination modality define the intrinsic optical response of the structure (Figure 5a): the hemisphere pole, which sets the axis of normal incidence; the hemisphere focus, located internally at half the radius and acting as a virtual convergence point for reflected rays under parallel illumination; the inflection band, where curvature and refractive-index contrast redistribute angular components; and the ellipsoid’s virtual focal curve, which governs the directional spread of reflected rays and shapes the emergent wavefront.

These geometrically defined features can be probed under different illumination conditions, without altering the underlying optical mechanism. Light–structure interaction can be decomposed into four limiting illumination regimes: (a) multiple point-like sources (Figure 5b), where each beam engages independently with curvature and lattice; (b) point sources whose reflected rays converge to a common focus (Figure 5c); (c) broad, uncollimated white-light illumination exceeding the heterostructure size (Figure 5d), where each surface element samples a distinct angular slice of the spectrum; and (d) partial transparency of both components (Figure 5e), allowing internal propagation and refraction. In all cases, reflection—and refraction in the fourth—are governed by the coupled action of local surface geometry and subwavelength periodicity. The illumination configuration therefore selects which aspects of the same geometry are emphasized, rather than introducing distinct optical regimes.

The following relations describe the local response to a single incident ray and therefore apply to any illumination that can be decomposed into angular components. Under these conditions, the standard planar Bragg condition

(1) $$m\lambda \;=\;2\;d_{hkl}\;\cdot \;n_{eff}\;\cdot \;cos\;\theta$$

no longer provides an adequate description, because both lattice orientation and surface normal vary continuously with position. Instead, it reduces to a local Bragg-type relation,

(2) $$m\lambda (r)\;=\;2\;d_{hkl}\;\cdot \;n_{eff}(r)\;\cdot \;cos\;\theta (r)$$

Continuous variations in curvature ensure that each surface region contributes a distinct combination of reflection and diffraction angles, producing spatially distributed spectral signatures rather than a single global photonic response [43,44]. Importantly, λ(r) does not vary arbitrarily. Because the surface normal evolves smoothly, θ(r) forms a coherent angular field, so λ(r) becomes a direct geometric mapping of morphology: hemispheres generate circular isochromatic contours, while ellipsoids produce elliptic ones, precisely as observed experimentally [31,32]. Curvature therefore organizes diffraction rather than randomizing it. In polar coordinates referenced to the pole, the hemispherical case satisfies θ(φ) = φ, yielding

(3) $$m\lambda (\varphi )\;=\;2d_{hkl}\;\cdot \;n_{eff}\;\cdot \;cos\;\varphi$$

For an ellipsoid with axial ratio c/a, the relation becomes

(4) $$m\lambda (\varphi ;\;c/a)\;=\;2d_{hkl}\;\cdot \;n_{eff}\;\cdot \;(cos\;\varphi /c)/sqrt[({sin}^{2}\;\varphi )/a^{2}\;+\;({cos}^{2}\;\varphi )/c^{2}]$$

Thus, curvature preserves the single-parameter nature of Bragg selection. For both hemispheres and ellipsoids, λ(r) reduces to λ(φ), directly analogous to λ(θ) in planar systems. This dimensional reduction explains the coherence of the observed isochromatic patterns despite the loss of global translational symmetry, and provides a direct route for geometrically programming spectral response through shape alone.

Within this geometry-defined framework, broadband white-light illumination reveals two additional optical phenomena: (i) a narrow, laterally collimated blue beam emitted perpendicular to the optical axis (Figure 5f), and (ii) displaced, ghost-like secondary foci when the structure is positioned outside the focal region of a converging lens (Figure 5g). Notably, neither the laterally collimated beam nor the ghost-like foci are observed in isolated hemispherical, ellipsoidal, or single-domain photonic crystals; their emergence requires the coupled action of curvature and internal interfaces within a single continuous heterostructure. The blue collimated beam arises from the coupling of local Bragg extraction within inverse-opal domains—selectively reinforcing wavelengths near the (111) stop band (~490 nm)—with curvature-induced refraction at locally cylindrical boundary regions. Broadband light undergoes spectral selection within the lattice, while the curved boundary redirects the selected component into a narrow lateral beam. Despite its simplicity, this effect—arising from the geometric projection of internally reflected ray families—has received little explicit experimental attention, although it is readily reproducible even with a classroom laser pointer, where near-normal illumination of a glass cylinder produces a lateral beam at 90° to the incident direction (Figure 5h,i). The photonic heterostructure reproduces this geometry while introducing wavelength selectivity through its internal lattice, transforming a purely geometric effect into a spectrally confined optical emission. The ghost-like foci arise from the same curvature-mediated redistribution of optical paths, reflecting nontrivial phase accumulation across coupled domains. These observations identify a transitional regime in which curvature reorganizes optical pathways while long-range order still governs spectral selection. Unlike geometry-driven effects engineered through refractive-index landscapes in top-down nanophotonics [45,46], the curvature here is intrinsic and imposed directly by the three-dimensional geometry of the self-assembled structure.

### 3.3. Coupled Order and Randomness in Light Interaction

The phenomena discussed so far arise in structures where curvature reorganizes light while long-range periodicity still governs spectral selection.

**Figure 6:**
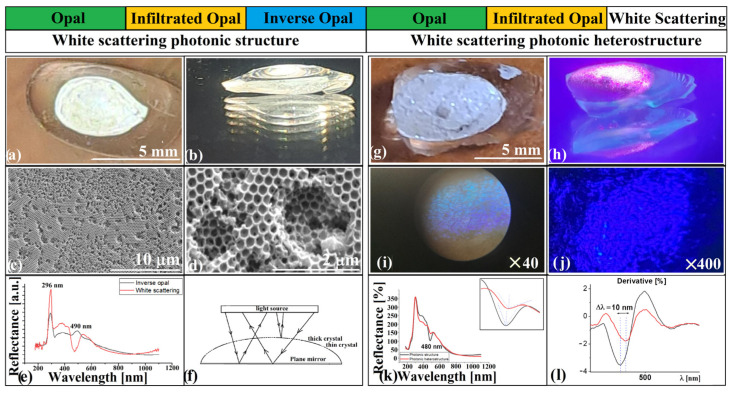
(**a**) Convex white-scattering structure; (**b**) Mirror multiple reflections; (**c**,**d**) SEM of white scattering metasurface; (**e**) Spectra of inverse-opal and white-scattering surfaces; (**f**) Light pathway schematic; (**g**,**h**) Illumination with white light and blue laser; (**i**,**j**) Microscopy through the pole; (**k**,**l**) Retro-reflection spectrum (with 480 nm zone inset zoom) and derivative.

Pushing the same fabrication route beyond this regime—by deliberately degrading periodicity through cyclic processing or embossing—reveals a second limit, where geometry becomes the primary organizing principle of light. In these white-scattering metasurfaces, disorder does not suppress coherence; it redistributes it along geometry-defined optical paths, producing deterministic spectral valleys rather than diffuse attenuation. As previously noted, a silica opal self-assembled by the hanging-drop method exhibits a high-quality interface at the air side and a more disordered interface at the substrate. To improve this lower side, a cyclic process is devised in which a silica opal formed on an ellipsoidal polystyrene support (Figure 6) is infiltrated with the substrate material and transformed into an inverse opal.

This inverse opal then serves as the substrate for a new cycle of opal formation, infiltration, and silica removal. Instead of a more perfect inverse opal, the process yields a surface with strong white-scattering character (Figure 6a). An unusual feature of this structure is its multiple-reflection behavior in a planar mirror (Figure 6b): when viewed near tangentially, it produces numerous, intense reflections of comparable size, an effect not observed in other translucent solids or conventional photonic crystals. SEM imaging shows a high density of micrometric “defects” (Figure 6c) within an otherwise well-ordered convex inverse-opal layer. Higher-magnification views (Figure 6d) reveal that each defect is a shallow concavity, 1–2 layers deep, that preserves local ordering and continues the global periodicity of the convex film without visible dislocations. The UV–Vis retro-reflection spectrum of this curved photonic structure (Figure 6e) does not exhibit a Bragg peak; instead, it presents a narrow valley located near the (111) stop-band position of a standard inverse opal. This valley arises from a geometry-driven redistribution of light rather than from simple suppression of reflection. White light enters preferentially through thinner concave zones of the metasurface (Figure 6f), reaches the interface with the bulk polystyrene, and is redirected back toward the surface. On the return path, a substantial portion of this light traverses thicker inverse-opal regions, which extract the Bragg-matched blue component laterally and remove it from the retro-reflected channel. The detector therefore registers a spectrum in which the Bragg-matched blue component is missing, producing a clean transmission-derived valley. In this configuration, curvature acts as an optical organizer. It defines distinct local pathways for injection, internal reflection, and spectral selection, while the white-scattering metasurface functions as a semi-transparent photonic filter whose response is determined by local photonic thickness. The behavior resembles an interferometric regime controlled not by planar cavity spacing, but by curved geometric pathways that allow extraction of the Bragg component and return of its complementary spectrum to coexist within the same structure. Although conceptually reminiscent of valley photonic crystal (VPC) filters in flat platforms [47] that achieve narrowband suppression via topological edge states and high-Q resonances, the present system operates through macroscopic geometric interference in a self-assembled, curved architecture. Both approaches aim at spectral engineering, but they rely on fundamentally different physical routes.

When the white-scattering metasurface is formed on a shallow spherical cap of polystyrene, the resulting heterostructure—comprising the metasurface and underlying convex inverse-opal mirror—exhibits a diffuse broadband reflection that remains strikingly white (Figure 6g). Under focused blue-laser illumination (λ = 405 nm, P = 1 W), the shallow cap reflects a partially specular beam that appears desaturated and rose-white (Figure 6h). The apparent loss of coherence does not originate from random scattering but from phase redistribution across the curved multilayer interface. Each local region of the metasurface–mirror pair acts as a microcavity with a distinct optical path length, and their superposition produces a diffuse yet spectrally preserved reflection. The process is therefore one of coherent averaging in a curved phase space, where geometry defines the range of allowed interference states. Reflection microscopy (Figure 6i,j) reveals two partially overlapping chromatic channels: a blue band arising from direct Bragg diffraction in the upper inverse-opal domains, and a magenta band corresponding to white light that enters through thinner concavities, is reflected by the convex infiltrated-opal mirror, and returns through the metasurface after selective removal of the blue component. Magenta thus represents the complementary spectrum of blue-filtered white light. The human visual system readily perceives both channels simultaneously because it reconstructs color from relative spectral deficits, while the spectrometer records only absolute intensities and reports the complementary contribution as a transmission-like valley centered near the Bragg wavelength. The spectral response (Figure 6k) confirms the coexistence of two distinct optical pathways: a retro-transmitted component forming the valley near 490 nm, corresponding to the (111) Bragg wavelength, and a geometry-dependent modulation introduced by the convex inverse-opal mirror, which generates a measurable asymmetry in the valley profile (Figure 6l). This asymmetry reflects redistribution of optical paths imposed by curvature: an interferometric regime in which geometry, rather than planar spacing, controls coherence organization. A conceptual parallel exists with flat metasurface platforms where optical functions are dispersion-engineered over broad spectral and angular ranges [47], yet the present curved system achieves a related spectral selectivity using macroscopic geometric injection, internal reflection, and Bragg-selective extraction instead of nanostructured phase programming.

**Figure 7:**
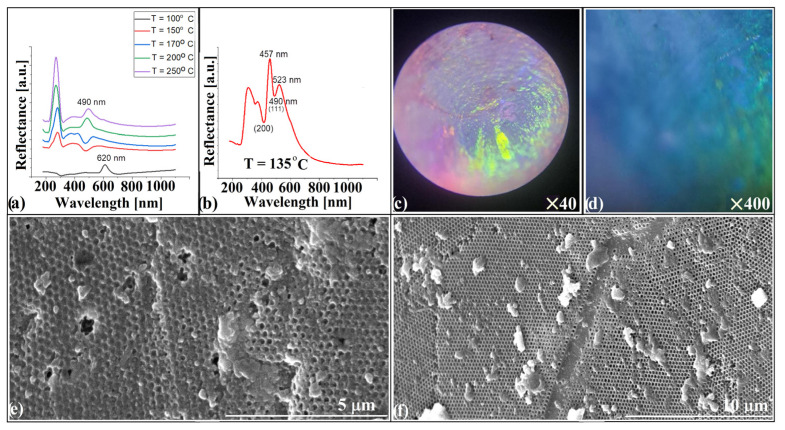
(**a**,**b**) Retro-reflection spectra of heterostructures synthesized at different synthesis temperatures; (**c**,**d**) Optical microscopy of embossed metasurface; (**e**,**f**) SEM of embossed surface.

Retro-reflection spectra of heterostructures similar to that in Figure 6k were obtained before the white-scattering effect was formally recognized. Samples synthesized at different temperatures produced UV–Vis spectra that, when superimposed (Figure 7a), revealed a systematic evolution. At high synthesis temperatures (250 °C and 200 °C), the inverse-opal metasurface is thick and well infiltrated, and only the reflection band of the (111) planes is visible around 490 nm. At lower temperatures (170 °C and 150 °C), valleys appear at the same position, most likely because fewer layers remain infiltrated or intact. At 100 °C, where infiltration ceases, the unstructured polystyrene becomes transparent and the reflection band of the underlying convex opal at 620 nm becomes visible.

At 135 °C, the metasurface reaches the threshold where white scattering first appears, initiating the 135–170 °C regime of disorder-driven broadband response. The retro-reflection spectrum displays a valley at 490 nm, interpreted as transmission through the (111) planes, flanked by two peaks at 457 nm and 523 nm—a distribution typical of single- or bilayer structures. A second valley overlaps the position usually assigned to (200) planes, and a third weaker one appears where no family of planes is expected to contribute. Optical microscopy (Figure 7c,d) shows the absence of the blue reflection usually associated with (111) planes in a polystyrene inverse opal with 264 nm voids, but is otherwise unremarkable. SEM images (Figure 7e,f) reveal alternating domains: hcp-like packing of partially embossed voids and one-layer inverse-opal regions. This morphology behaves as a white-scattering surface similar in effect to that produced by cyclic synthesis, but with a distinct spectral fingerprint. Although the inverse-opal metasurface contains only one to two photonic layers, its curvature embeds it in a three-dimensional optical environment. As a result, the system ceases to behave as a planar 2D photonic lattice. The continuously varying surface normal enforces angle-averaged Bragg sampling, while the underlying convex substrate provides effective optical depth through multiple internal reflections. Together, these effects cause a curved 2D layer to be perceived by light as a shortened three-dimensional crystal, preserving the spectral signatures of the (111) and (220) families even in the absence of true 3D periodicity. The remarkable agreement between experimental valleys and FCC stop-band positions therefore reflects a geometry-mediated 2.5D photonic response rather than conventional Bragg diffraction. Surprisingly—or perhaps inevitably—this type of metasurface architecture, consisting of alternating hcp single-layer domains and mechanically embossed regions, is entirely absent from a recent programmatic roadmap on photonic metasurfaces [48]. The authors focus almost exclusively on top-down fabrication routes and their associated phase-engineered planar geometries, overlooking structurally heterogeneous, self-assembled, and curvature-compatible configurations such as those demonstrated here. More importantly, the roadmap itself acknowledges a critical limitation of the field: “…the field lacks methods to capture distributed coherence in curved or mesoscale architectures… present numerical and experimental approaches assume planar boundary conditions” [48]. The metasurface presented in Figure 7 directly occupies this missing conceptual space. Rather than enforcing planarity, it operates through geometry-induced variation in photonic thickness, distributed coherence, and defect-assisted spectral selection—all phenomena that lie outside the design rules of flat metasurfaces and are not accounted for in current theoretical frameworks. As such, it represents a complementary pathway for metasurface engineering, one that emerges naturally from self-assembly and curvature rather than from lithographic phase programming. Although the present work focuses on structural and phenomenological characterization, further quantitative analysis—particularly spatially resolved optical measurements—would provide deeper insight into the underlying mechanisms.

However, the modest apparent magnitude of the optical effects arises not from weak phenomena but from the limitations of instruments designed for planar samples. In curved photonic systems coherence and spectral selection are redistributed along multiple coupled trajectories; planar tools therefore suppress or erase the information they are not built to capture.

### 3.4. Limitations and Potential Applications

The present approach is subject to several practical limitations that define its current scope. First, the range of accessible geometries is largely restricted to convex structures, as dictated by the hanging-drop configuration. Extending the method toward concave or more complex geometries remains a significant challenge and would require alternative strategies for curvature control and stabilization.

A second limitation concerns the range of materials. The current implementation relies primarily on polystyrene due to its favorable thermal and rheological properties. Expanding the method to other polymers or material systems with different optical and mechanical characteristics, including higher refractive index systems, would significantly broaden its applicability.

In addition, the investigation of curved photonic structures exposes fundamental limitations of conventional characterization techniques, which are predominantly designed for planar systems. The development of experimental approaches capable of resolving spatially and angularly dependent optical responses on curved surfaces remains an important direction for future work. The scalability of the fabrication process toward high-throughput production of functionally consistent curved photonic structures, rather than strictly identical replicas, also remains to be addressed.

Despite these constraints, the demonstrated control over curved photonic heterostructures reveals optical functionalities that are inherently inaccessible to planar architectures. In particular, curvature introduces a spatially structured optical response, where propagation, reflection, and spectral behavior become position-dependent rather than globally defined. This enables effects such as wide-angle light manipulation and spatially varying spectral filtering within a single, integrated structure. From an applied perspective, the free-standing and mechanically robust nature of these structures facilitates handling and integration into practical configurations, overcoming the fragility and alignment sensitivity typically associated with planar photonic crystals. Moreover, their operation relies on passive, geometry-driven mechanisms, enabling optical functionality without external power input or active control.

Two distinct regimes govern the coupling between geometric and wave-optical descriptions in these systems. In the first, geometry acts as a passive mediator, simultaneously constraining ray trajectories and phase evolution. In this regime, curved photonic structures may be employed in position-sensitive optical sensing, wide-angle light manipulation, and spatially varying spectral filtering, enabling compact optical elements with geometry-dependent response.

In the second regime, an active coupling emerges in systems supporting multiple internal reflections and partial transmission. Here, ray-defined propagation paths generate repeated interference and phase accumulation, allowing the structures to function as distributed optical cavities with geometry-controlled spectral response. This behavior may be exploited for spectral shaping, emission control, and compact photonic elements operating on non-planar geometries.

More generally, these results point toward a class of optical architectures in which geometry defines a functional mapping between input and output signals. Such systems enable intrinsic encoding of optical information through curvature, opening new approaches to sensing, light manipulation, and spectral processing beyond the limitations of planar designs.

An additional perspective arises from the controlled interplay between ordered and disordered regions engineered within these systems. As discussed in Section 3.3, the coexistence of structural order and controlled disorder can give rise to hybrid optical responses that combine coherent and diffuse scattering. This suggests that curved photonic heterostructures may provide a platform for exploring photonic states intermediate between crystalline order and fully disordered media, such as photonic glass-like behavior or disorder-assisted light manipulation.

Such regimes may enable functionalities that are not accessible in purely ordered systems, including enhanced scattering control, isotropic optical responses, or broadened spectral features arising from the coupling between periodic and aperiodic domains.

Importantly, the role of curvature in this context extends beyond geometric shaping. Curvature inherently disrupts global translational symmetry, leading to the emergence of spatially varying local order and unavoidable structural defects. As a result, ordered and disordered regions are not spatially separated, as in planar systems, but become intrinsically coupled within the same architecture.

This geometry-imposed coexistence enables optical responses that combine coherent (Bragg-like) and diffuse (scattering-dominated) contributions, giving rise to hybrid regimes that cannot be realized in flat photonic structures. In this sense, curvature acts as a mediator that enforces interaction between order and disorder, rather than allowing them to exist as independent regimes.

## 4. Conclusions

Beyond demonstrating curvature-driven optical effects, the present results highlight the versatility of polymer-based photonic architectures. The use of molten polystyrene as both structural matrix and optical medium enables the fabrication of millimetric curved inverse-opal systems that remain inaccessible to conventional planar polymer photonic platforms.

The central result of this work is that geometry acts as an active degree of freedom that selects physically accessible optical states, revealing modes that planar architectures systematically suppress. A synthesis of the experimental results and the conceptual framework developed in this study reveals a set of causal links that distinguish the photonic behavior of curved heterostructures from that of planar systems. These links organize naturally into the following hierarchy:

Geometry-Induced Photonic Consequences:

Primary effects (directly imposed by curvature):

- Spatially varying local normal;
- Non-parallel lattice orientations;
- Position-dependent incidence angles and optical axes.

Secondary effects (emerging from primary geometry):

- Topological defects (geometrically unavoidable);
- Frustrated coupling between 2D and 3D symmetries;
- Loss of global translational symmetry; formation of local symmetry patches.

Tertiary effects (arising from secondary structure):

- Distributed rather than uniform coherence;
- Path-dependent phase accumulation;
- Local expression of wave–particle duality;
- Optical and quantum modes accessing an expanded, geometry-conditioned state space.

Final consequence:

- Photonic behaviors fundamentally inaccessible on planar substrates.

While the present framework emphasizes geometry-induced ordering and its optical consequences, it is important to note that partial disorder, structural defects (e.g., voids and vacancies), and dielectric inhomogeneities may further modulate the optical response.

### Outlook Symmetry and the Hidden Degrees of Freedom of Light

Current photonic theories describe light only through the restricted subspaces their mathematics can accommodate. Interference optics, geometric optics, quantum optics, and band-structure theory are not competing explanations but selective projections: each formalism isolates a single operational slice of a physical entity whose full behavioral spectrum exceeds any individual theoretical frame [49,50]. What we interpret as the nature of light is therefore, to a large extent, the nature of the constraints imposed by our theoretical lenses. Curved photonic architectures expose precisely those modes that planar formalisms suppress, revealing portions of light’s potential that remain inaccessible in flat geometries.

This difference becomes evident when identical building units assemble on planar versus curved substrates. In planar systems, local order extends seamlessly into global 2D and 3D order through perfect translational symmetry [1]. A single surface normal defines a unique optical axis, and symmetry propagates uniformly across the entire structure. Curved systems break this determinism. On a curved surface, 2D order remains strictly local, and any attempt to extend it requires topological defects and geometric frustration, as established in the theory of spherical crystallography and curved-lattice packing [51,52,53]. The coupling between 2D and 3D symmetries becomes position-dependent, variable, and non-translational; structural invariants, not imperfections. They enforce a topologically mandated symmetry landscape that planar systems cannot realize. Optical modes inherit this fragmented symmetry: coherence becomes distributed, phase accumulation becomes path-dependent, and the system supports regimes that planar detection geometries cannot register [54]. This is because the distinction between planar and curved photonic systems is not purely geometric but topological. Any two-dimensional lattice embedded on a curved surface must generate unavoidable topological defects; they are structural invariants, not imperfections. Their presence partitions symmetry into local domains, and optical modes encode this partitioning. Curvature therefore does not merely distort a planar lattice; it enforces a topologically mandated symmetry landscape that planar systems cannot realize. Spatially varying surface normals impose correlated phase relationships even when periodicity is fragmented. This geometric coherence arises from constrained phase propagation on curved manifolds, enabling modes that remain coherent despite reduced structural perfection. This altered symmetry reframes the wave–particle duality. In conventional optics, duality appears as a binary contrast: delocalized interference versus localized detection. But this dichotomy reflects the geometry of the instruments rather than an intrinsic property of light. Planar systems enforce global translational symmetry, collapse the accessible mode space, and suppress intermediate regimes between wave-like and particle-like behavior. Light “chooses” a manifestation only because the geometry eliminates the continuum between them. Curved photonic architectures restore that continuum. Spatially varying normals and frustrated symmetry domains allow localized, particle-like energy pockets to coexist with extended, wave-like phase correlations. Duality becomes geometry-dependent: planar geometries reveal only the endpoints; curved geometries reveal the spectrum between them. Recent work on curvature-induced wave localization supports this reinterpretation [55]. Curved photonics does not create alternative physics; it uncovers degrees of freedom that planar optics systematically projects out.

If curvature reveals optical modes absent from planar architectures, the quantum extension follows naturally. Hilbert-space invariance holds only when geometry does not constrain the admissible states. In flat space, the Schrödinger equation contains no geometric terms,

(5) $$-(\hbar^{2}/2m)\;\nabla^{2}\;\psi \;=\;E\;\psi ,$$

and a single surface normal defines a single class of admissible modes. The Hilbert space remains geometry-invariant precisely because the underlying Euclidean geometry imposes no restrictions on symmetry or admissible states.

Introducing curvature replaces the Euclidean Laplacian with the Laplace–Beltrami operator plus the geometric potential (H^2^ − K), the standard result of quantum confinement on curved manifolds [56,57,58]. This modification is the quantum analogue of replacing a global Bragg angle with a local one: geometry becomes an active selector of modes,

(6) $$-(\hbar^{2}/2m)[∆\Sigma \;\psi \;+\;(H^{2}-K)\psi ]\;=\;E\;\psi ,$$

On a sphere, where curvature is constant, the equation simplifies and the spectrum becomes discrete,

(7) $$-(\hbar^{2}/2mR^{2})L^{2}\;\psi \;=\;E\;\psi ,$$

a structure incompatible with the continuous plane-wave basis of flat space. No unitary transformation preserves the symmetry structure needed to map plane-wave modes onto spherical-harmonic modes, since the relevant symmetry groups (translations vs. SO(3)) are not isomorphic [59,60].

On an ellipsoid, curvature varies across the surface and symmetry becomes fractured. The governing equation acquires fully position-dependent coefficients,

(8) $$-(\hbar^{2}/2m)[{∆}_{ellip}\;\psi (\varphi ;\;c/a)\;+\;(H^{2}(\varphi ;\;c/a)\;-\;K(\varphi ;\;c/a))\psi (\varphi ;\;c/a)]\;=\;E\;\psi (\varphi ;\;c/a),$$

a quantum counterpart of the curved Bragg relation (4), where geometry explicitly determines the admissible modes and no global symmetry remains to enforce spectral uniformity [61].

Equations (5)–(8) express a single principle: geometry is not a passive coordinate choice but an active selector of quantum states. Planar and curved quantum systems do not share the same configuration-space Hilbert representation or symmetry class, and therefore support different physical modes. Geometry does not merely remap a fixed basis—it changes the set of physically realizable states. Quantum interference, phase accumulation, superposition structure, and noise coupling all depend on the symmetry group of the configuration space [59,62]. On planar substrates, these symmetries are translational and separable; on curved architectures, they are local, frustrated, and topologically constrained. As a result, curved systems can stabilize interference pathways that decohere on planar surfaces, suppress noise channels that require extended symmetry, and generate new quantum transport modes shaped by curvature-induced coherence redistribution [55]. Seen in this light, curved quantum systems are not geometric variants of planar ones; they are qualitatively different quantum objects.

The emergence of non-planar optical modes in classical photonics is the experimental footprint of this deeper principle. Curved quantum architectures may therefore inherit an expanded state space, providing a route toward quantum devices naturally resistant to noise, temperature, and disorder, echoing early proposals on geometry-conditioned decoherence suppression [63].

Taken together, these arguments support a single proposition: the geometry of a photonic system is not a passive backdrop but a determining variable that selects which subset of light’s unrealized potential becomes observable. Planar systems reveal only what their symmetry allows; curved systems widen the accessible domain. Geometry does not modify light—it reveals it.

The broader implications extend beyond photonics. In white-scattering domains, where order and disorder coexist, coherence is not lost but redistributed. Optical behavior arises not from ideal periodicity but from geometry’s ability to sustain coherence under constraint. This suggests a deeper principle: the capacity of structure to inherit stability without replication. This may have operated long before biological systems emerged—in the prebiotic interplay of light, minerals, and fluids. Within curved and partially ordered environments, such as droplets, colloidal domains, or natural opals, order and diffusion coexist naturally, allowing transient but coherent patterns to emerge and persist. Before the appearance of genes or membranes, geometry itself may have been the first architect of complexity—a silent teacher that showed matter how to remember. The energetic and entropic cost of building order from chaos is immense; yet borrowing it from preexisting crystalline or photonic templates is minimal. Opaline materials, in particular, concentrate, reflect, and recycle sunlight, creating localized electromagnetic fields that could have enhanced photochemical reactions and selective bonding [49,64,65]. Their surfaces, where ordered regions blend with gentle disorder, form interfaces where coherence meets fluctuation—natural incubators for early cooperative molecular networks. From this perspective, life did not arise as a rupture in the inorganic continuum but as its most refined modulation, a resonance between light, symmetry, and matter. The earliest living systems may not have invented order; they inherited it from structures that had already learned to trap and sculpt light.

Just as opaline architectures may once have nurtured the origins of life, modern curved photonic heterostructures could seed technologies yet unimagined. The future of photonics may depend not on flawless periodicity but on spontaneous symmetry, cooperative emergence, and curvature—signatures of freedom where control and spontaneity coexist and where light and matter collaborate rather than compete. In this sense, beauty becomes the final criterion of experiment: the point where understanding and form converge.

## Data Availability

The original contributions presented in this study are included in the article. Further inquiries can be directed to the corresponding authors.
